# New products

**DOI:** 10.1155/S1463924686000202

**Published:** 1986


Journal of Automatic Chemistry/*Journal of Clinical Laboratory Automation*, Vol. 8, No. 2 (April–June 1986), pp. 90–100

### New products

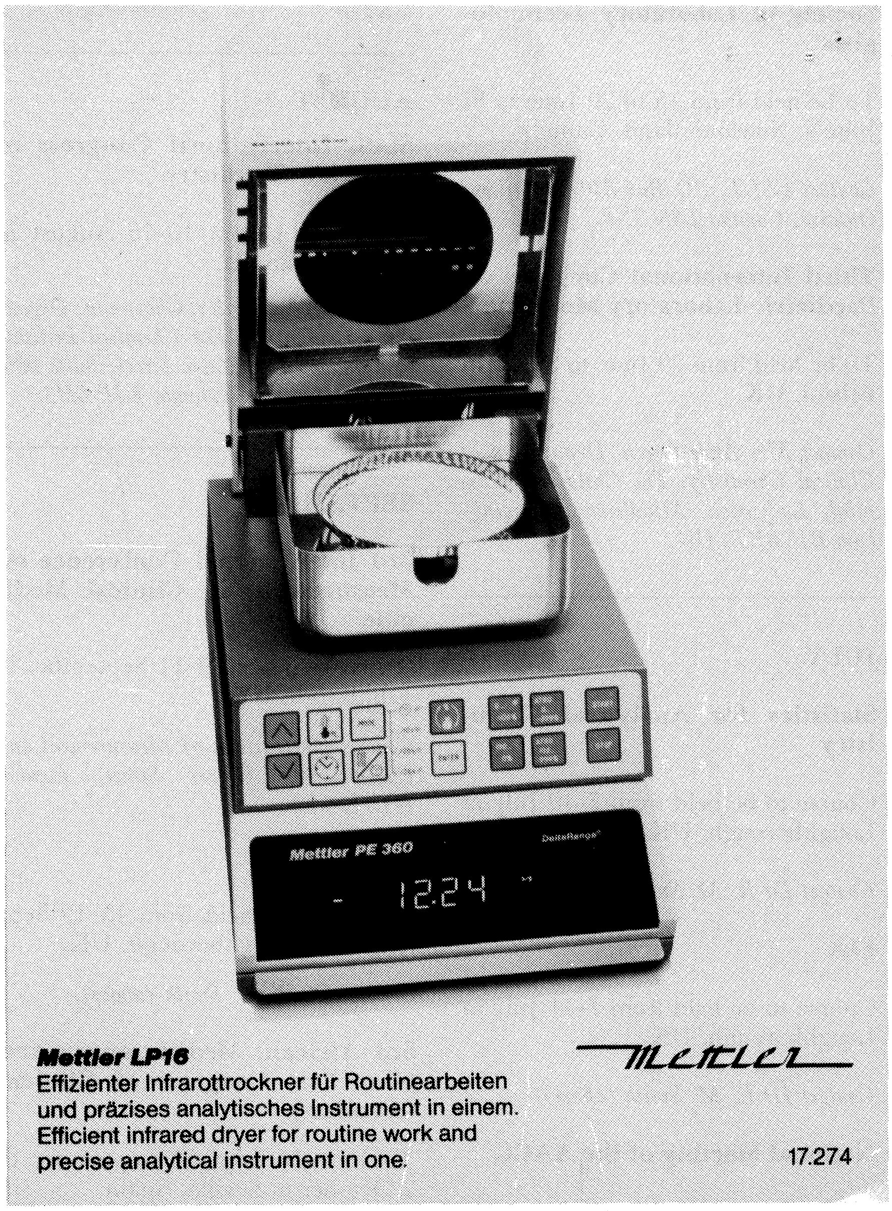

*Mettler's LP16 is an infra-red drying unit for routine determinations of moisture and dry weight in production control. It performs drying cycles automatically, with every aspect closely controlled. The LP16 meets the exacting requirements of modern quality assurance laboratories—it analyses any sample precisely, under reproducible drying conditions and easily configured for specimens of all kinds. The drying temperature is set in advance: two built-in sensors continually measure and regulate the temperature, so controlling the drying sequence. The infra-red drying unit will heat for a chosen length of time; determinations to a definable dryness end-point can also be configured. There are four different evaluation programs for computing the results. The LP16 is used in conjunction with a Mettler precision balance, although the two can be disconnected very quickly. The balance can then be used quite normally for weighing. Like all Mettler instruments, a standard feature of the LP16 is a data interface for connecting to a printer thus providing automatic, detailed records. For more extensive data processing the infra-red drying unit can link up to computers as well. Details from Mettler Instruments Ltd, Kingsmead House, Abbey Barn Road, High Wycombe, Buckinghamshire HP11 1QW, UK. Tel.: 0494 450202.*

**Circle No. 31 on Reader Enquiry Card**

### HPLC

Designed for the routine production quality control laboratory, Beckman's QC/Iso System is a low-cost, low maintenance workhorse system that is simple to operate without special training, yet is easily automated for unattended operation. This high performance liquid chromatography system provides fast, reliable, reproducible results for quality control chemists particularly in pharmaceutical, food and beverage, brewing, cosmetic and agricultural industries.

The QC/Iso system is pre-plumbed and easily customer-installed. The package is configured so that individual components can be seen and accessed easily, enabling easy maintenance and service.

Time-tested components of the system—the Beckman Model 110B pump, Model 160 fixed wavelength UV detector and the Model 210A injection valve—offer excellent reliability. Because of a unique slide-mounted injection valve, the system can accommodate any HPLC column from 45 to 300 mm long without loss of chromatographic integrity, providing users with the choice of optimising the separation for speed, resolution or sensitivity.

*Details from Beckman Ltd, Progress Road, Sands Industrial Estate, High Wycombe, Buckinghamshire, UK. Tel.: 0494 41181.*

**Circle No. 32 on Reader Enquiry Card**

### Flat coreless motor

The Y704 Coreless DC Motor, which is 47 mm thick, is available in the UK exclusively through Drivematic Ltd. The Y704 is a 12 v d.c. motor of traditional pancake coreless construction, 150 mm in diameter. Under typical duty conditions the Y704 will deliver 3750 rev/min at 2200 g-cm torque, with input power rated at 140 W. It was originally designed for the automobile industry for such applications as radiator cooling fans and other similar uses where space is limited. Due to its compact frame size, the Y704 finds increasing accpetance in other fields such as medical instruments, computer peripherals and applications where space is at a premium.

Simple design and robust construction allow the Y704 to be offered at a highly competitive price, as low as £20 per unit for quantity orders.

*For further information contact Philip de Vall, Drivematic Ltd, 190 Weston Lane, Birmingham B11 3RX, UK. Tel.: 021707 3534*

**Circle No. 33 on Reader Enquiry Card**

### ICI signs laboratory computer software licensing agreement

Imperial Chemical Industries PLC (ICI) and Nelson Analytical, Inc. of Cupertino, California have signed a licensing agreement which gives the American company the right to market, support and develop further ICI's computer software for analytical chemistry laboratories. The software is a comprehensive Laboratory Information Management System developed by ICI's Agricultural Division at Billingham (UK), which utilizes the VAX and MicroVAX computers manufactured by Digital Equipment Corporation. Nelson Analytical specializes in computer software products for analytical laboratories.

David Nelson, President of Nelson Analytical, has said that the agreement would give his company a tested, complete product from a leading real-life user well ahead of the time they could have developed it themselves. The product will be launched commercially this Spring.

*For further information call (UK) 0642 522453.*

**Circle No. 34 on Reader Enquiry Card**

### Single instrument for TOC, TOD and TC

Ionics have introduced a microprocessor version for their Lab 1200 series of water analysers. Lab 1200 analysers have applications in chemical and food processing; pulp, paper and paint manufacture, pharmaceuticals and waste water treatment. Principal uses include the measurement of effluent quality, product losses and spill detection.

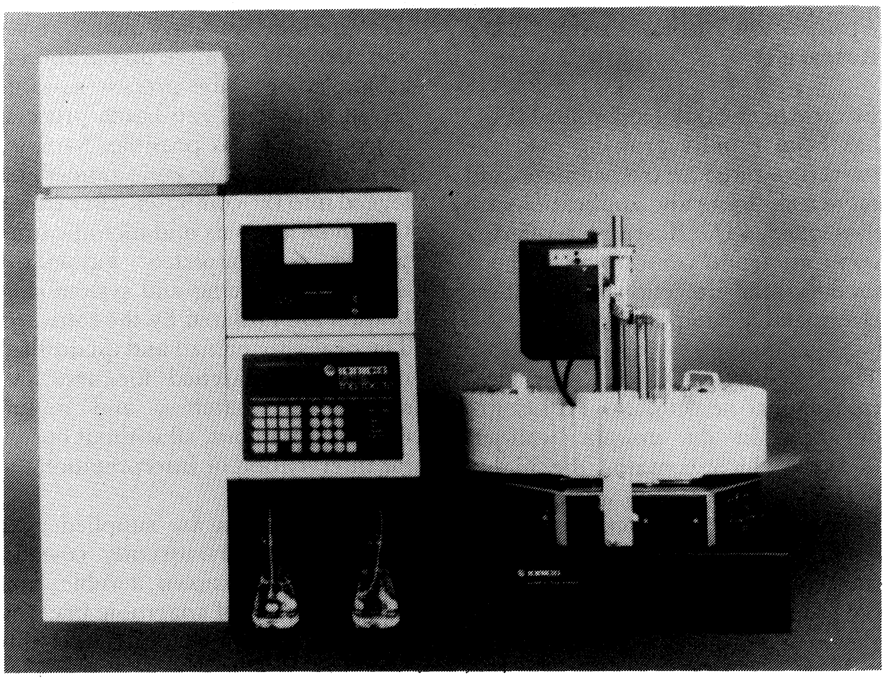

*The Ionics 1270M (see text). Other models in the Ionics 1200 Series include dedicated TC, TOC and TOD monitors used to measure organics in waste and drinking-waters, and in cooling and condensate/boiler feed water. They are simple to operate even when samples are affected by normally difficult conditions such as the presence of suspended and dissolved solids, turbidity and extreme pH.*

From a single sample injection, users of the new Ionics 1270M can obtain a complete wastewater profile to include TC, TOC and TOD in around 5 min. It can also measure inorganic carbon and purgeables including volatile insoluble organics which could be lost during carbonate removal processes.

Sample concentrations are continuously digitally displayed, as are diagnotices routines to monitor the status of critical system components. A print-out gives sample number, analysis performed, each concentration value and the average value for repetitive analyses.

Concentrations are automatically determined by both peak height and peak area; and an RS232C output enables users to interface to other laboratory computers.

1200 Series analysers have been specifically designed for general water analyses, including dirty water samples. They employ an all-ceramic automatic sampling valve to handle most dirty water applications, giving repeatable sample injections with little maintenance. A post furnace scrubber has been incorporated to collect waste material from the sample combustion thus reducing the time required to maintain the detector. Components are also designed to be easy to clean or change fairly quickly.

*For further details contact Ionics Inc., 10 Stratham Avenue, Lymm, Cheshire WA13 9NH, UK.*

**Circle No. 35 on Reader Enquiry Card**

### Automatic amino-acid analyser

Liquimat IV is an automatic amino-acid analyser using single column ion exchange resin technology. It was designed not only for routine analyses but also for medical research, microbiology, botany, pharmaceuticals and the food industry. Based on established HPLC techniques, the instrument uses post-column reaction with ninhydrin or OPA allowing detection by UV or fluorescence.

*For further information contact Radiomatic Instruments and Chemicals Ltd, 11 Lincoln Park Business Centre, Lincoln Road, High Wycombe, Berkshire, UK. Tel.: 0494 452216.*

**Circle No. 36 on Reader Enquiry Card**

### Application notes from EDT Research

Electrochemical detection for HPLC has been rapidly growing over the past two–three years as more and more techniques and applications are developed. EDT Research is offering application notes describing methods used for the detection of countless electroactive compounds.

The success of EDT's LCA 15 electrochemical detector for HPLC lies not only in the unique 'wall-jet' flowcell which contains the highly polished glassy carbon working electrode but also the excellent stable, drift-free electronics for the controller. This combination has made the LCA 15 the most popular electrochemical detector known.

The application notes available are:

(1) General chromatography suitable for electrochemistry
(2) Morphine
(3) Penicillamine
(4) Phenols
(5) Metal ions
(6) Amines.

*For free copies of these application notes contact EDT Research, 14 Trading Estate Road, London NW10 7LU. Tel.: 01 961 1477.*

**Circle No. 37 on Reader Enquiry Card**

### Automatic microbial identification system

A computer-controlled system from Hewlett-Packard provides an innovative, fast, cost-effective method of accurately identifying bacteria, yeasts, moulds and other microbes. Based on the high resolution gas-liquid chromatographic analysis of cell wall fatty acids which provide a characteristic 'fingerprint' of the microbial strain, the method utilizes advanced software which searches a library data-base of fatty acid profiles to identify the organism at genus, species and sometimes even sub-species level.

At the heart of the HP 5898A Microbial Identification System is the proven HP 5890A chromatograph, an automated unit which separates and quantifies the fatty acids quickly and precisely. Sample preparation is a simple, one-tube process suitable for all microbes, and batch processing of samples is possible. Samples are inserted into the autosampler and logged into the computer, after which the whole analysis and identification process is completely automatic. Sample sequencing and system calibration are initiated by the software. The peaks are named and quantified, the library searched for best and second-best matches, and a full report printed out, all without operator intervention or interpretation.

The library data are supplied with the system, and currently cover a wide range of known aerobic bacteria. Libraries of anaerobic bacteria and of yeasts will be added later this year.

With the new system, up to 50 microorganisms a day may be identified, at an operating cost that compares very favourably to conventional methods. Many microbes can be identified that are difficult to recognize by other techniques, and in most cases this can be done at a fraction of the cost. The HP 5898A Microbial Identification System will find applications in such areas as medical research, agriculture, pharmacology, many industrial processes, sterility control, water quality monitoring, and environmental analysis.

*Product enquiries to Tina Mears, Analytical Instrumentation, Hewlett-Packard Ltd, Miller House, The Ring, Bracknell, Berkshire RG12 1XN, UK. Tel.: 0344 424898.*

**Circle No. 38 on Reader Enquiry Card**

### Tapered multipurpose vials

A new vial design from Chromacol Ltd has been described as likely to become the industry standard. The design profile is that of a standard 2 ml vial (about 32 × 12 mm) but with a taper at the base. There are two options, the 1·1-CTV with a crimp-top and the 1·1-STV with a screw-top. Both types hold a maximum of about 1·1 ml. The minimum usable capacity will depend on individual instruments and needles. Side hole needles will require more volume than the conventional needles. By combining the general dimensions of the standard 2 ml vial (1·5 ml to some and 1 ml to others) many chromatographers will be able to standardize on one vial for runs where quantity is no problem as well as for micro-volume work.

*Information and prices are available from Chromacol Ltd, Glen Ross House, Summers Row, London N12 0LD. Tel.: 01 368 7666, or its authorized dealers.*

**Circle No. 39 on Reader Enquiry Card**

### Amino-acids

The system 7300 amino-acid analyser from Beckman features a special dual-channel data system than offers new capabilities for amino-acid analysis.

The system automatically labels each peak on the chromatogram with the name of the amino-acid, eliminating time-consuming correlations with elution times. It calculates and prints out both 440 and 570 nm colorimeter signals for greater quantitative accuracy at picomole levels. Its floppy disk stores up to 40 analyses and permits reassignment of base-lines and reformatting of data.

Similarly to the Beckman System 6300, the 7300 is a dedicated high-performance ion-exchange chromatograph featuring a single, prepacked stainless steel column operating at up to 3000 psi. It performs hydrolysate analyses in 30 min, and physiological analyses in 2 h. These results are guaranteed by Beckman, with detailed methods, applications back-up and full field service support.

*Details from Beckman Ltd, Progress Road, Sands Industrial Estate, High Wycombe, Buckinghamshire, UK. Tel.: 0494 41181.*

**Circle No. 40 on Reader Enquiry Card**

### BBC micro and Philips pH meter

The PW 9422, which heads Philips Analytical's latest range of pH meters, can be linked to a microcomputer such as the BBC B to meet operational and programming requirements.

The link-up also means that users can obtain ion-selective measurements, have extended use of ion-selective electrodes, and carry out a dialogue with the computer.

The PW 9422 itself is a microprocessor-controlled, push-button-operated pH meter. The microprocessor refines accuracy by calculating the pH value automatically and directly, eliminating any need to adjust potentiometers. Temperature compensation, too, is excellent in the PW 9422. The microprocessor simply computes changes in electrode slope with temperature, superseding the old method of adjusting analogue circuitry.

Autocalibration is another microprocessor benefit, doing away with the need for entering buffer values and so streamlining operating technique. Autocalibration identifies the NBS buffer value and automatically uses its temperature-adjusted value during calibration.

The PW 9422 provides a 4½ digit LED display of pH, mV or temperature, with discriminations of 0·001 pH, 0·1 mV or 0·1°C over the complete range. A digital RS232C output enables the meter to be connected directly to a printer or microcomputer.

*More information from Pye Unicam Ltd, York Street, Cambridge CB1 2PX, UK.*
**Circle No. 41 on Reader Enquiry Card**

### UV/Vis scanning spectrophotometers

Philips Analytical has introduced a new range of VDU-based, mid-range, ultra-violet/visible scanning spectrophotometers—the PU 8820 series. They are based on the PU 8800 UV/Vis instruments and have been priced at a level to suit the more budget-conscious laboratory organization.

The new spectrophotometers provide an outstanding range of standard features, including log A, first to fourth derivative and derivative concentration for wavelength scanning, absorbance and %T.

In fixed wavelength, results are presented directly in concentration, complete with user-defined units and a wide range of enzyme analysis routines.

With its built-in VDU, the PU 8820 series is particularly easy to operate. The supportive user interface, which offers operational prompts, audio-visual error warnings and two-key parameter selection, provides both fast and confident operation, irrespective of skill level.

Performance has been optimized for routine applications, and features such as master holographic gratings, silica coated optics and a very large, totally sealed sample compartment, combined with resolution to 0·1 nm, guarantee top quality analytical data.

An extensive range of accessories ensures that PU 8820s can be adapted quickly, conveniently and effectively for a variety of applications.

There are two versions in the series. The PU 8825 includes a built-in recorder and electrostatic printer and is recommended for routine, stand-alone operation, while the PU 8820 has been developed for users who want to connect their own recorder or have a current or future requirement for computer operation.

With its standard bi-directional RS232C interface, the PU 8820 has been optimized for interfacing to the recently announced, IBM environment, Philips Analytical data station.

*Further information from Pye Unicam Ltd, York Street, Cambridge CB1 2PX, UK. Tel.: 0223 358866.*
**Circle No. 42 on Reader Enquiry Card**

### Data-handling system cracks IR problem

A method which overcomes the common problem in infra-red spectrophotometry of the quantitative analysis of one component in a mixture has been developed by Philips Analytical. The system employs the company's new IBM PC environment data station and an integrated data system for IR spectrophotometry which is based on Philips Analytical's PU 9500 or SP3 series of ratio-recording instruments. High-resolution colour graphics, speed and memory size are utilized to provide accuracy, ease of use and versatility.

The analysis of one component in a mixture has been a longstanding headache in organic chemistry, made even more difficult when the spectra of the constituents are very similar.

At Philips Analytical's applications laboratory in Cambridge, the assignment was to assess the trans-isomer content in a cis- and trans-methyl ester mixture. Comparison of the spectra of the two pure compounds as 2% solutions in carbon disulphide showed them to be extremely similar, although the trans-isomer had a strong band at 966 $cm^{-1}$ whereas the cis-isomer only weakly absorbed in this region.

After the cis-isomer component had been removed by spectral subtraction, the band was used to determine the concentration of trans-methyl ester in a mixture by comparing peak height with a standard. As cis-isomer absorbs in this region it is important that it should all be removed from the mixture in this way before measurement.

Since all cis-methyl ester peaks coincide with those of the trans-isomer, it is not possible for the computer to use any of them to calculate the factor required to reduce the cis-isomer peaks to zero upon subtraction from a spectrum of the mixture.

Using the peak at 966 $cm^{-1}$ the computer automatically calculates the factor to enable trans-isomer peaks to be reduced to zero on subtraction from a spectrum of the mixture. The resultant spectrum refers to the cis-isomer component only.

Subtraction of this spectrum from thaty of the original cis- and trans-isomer mixture leaves that of the trans-isomer component in the mixture, which can be quantitatively measured using the 966 $cm^{-1}$ peak.

This method can be stored as a sequence in the computer, enabling the user to carry out a complete analysis on a mixture using a single command.

*Further information from Pye Unicam Ltd, York Street, Cambridge CB1 2PX, UK. Tel.: 0223 358866.*

**Circle No. 43 on Reader Enquiry Card**

### Electric motor design

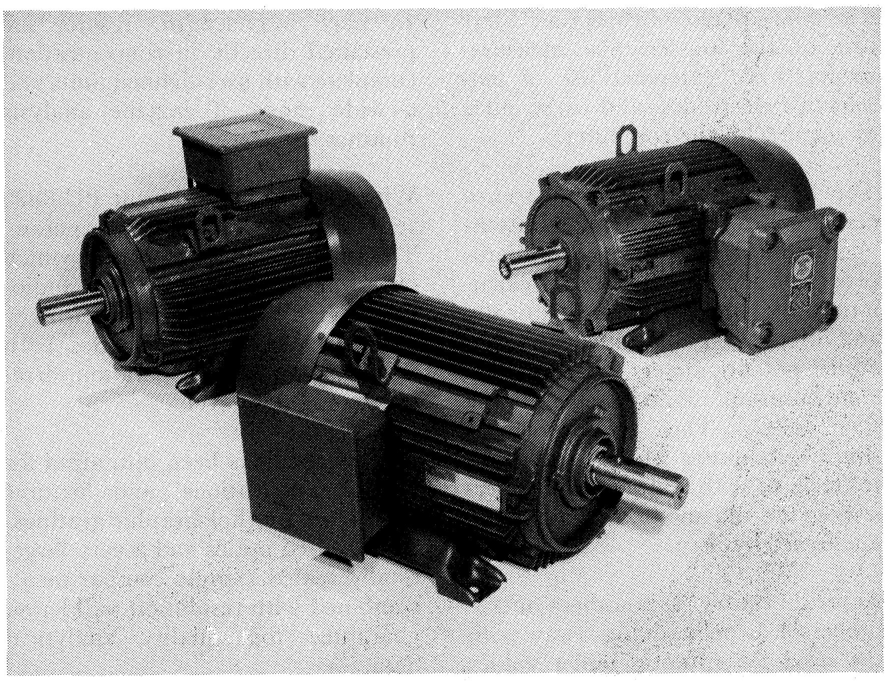

*The first family of three motor types in Newman's new 'MM Series' electric motor design. In the foreground is a lightweight, alloy-framed TEFV version. Behind are the two cast-iron framed Hi-Seal machines with upgraded enclosure for harsh environments; the GP (left) and the EExd flameproof versions.*

An entirely new design of three-phase AC induction motor, weatherproof and hoseproof to IP55 and said to give unprecedented choice of enclosure and instant adaptability to almost any industrial application, has been launched by Newman Electric Motors.

The 'MM Series' is based on a modular form of construction, applied to motors constructed either in lightweight high-tensile alloy extruded frames, or in cast-iron.

Removable pressed-steel feet, together with add-on adaptor rings for flange or face mounting and other features, give stockists and end users nearly 600 options from the one basic design. And many of these choices, according to Newman, can be made instantly, by simple adaptation of off-the-shelf stock motors.

There are four basic standard enclosures in the series: a standard TEFV (totally enclosed fan-ventilated) in the alloy frame, and three in cast-iron frames to Newman's up-graded enslosure standard, known as 'Hi-Seal'. The MM Series Hi-Seal will carry a two-year guarantee. The three 'MM' Hi-Seal categories are: the 'GP' for general purpose duty in wet, dusty or otherwise harsh environments; the similarly protected but also ExN certified spark-proof type for semi-hazardous locations; and the EExd 'flameproof' type for hazardous environments.

Though the standard MM Series TEFV motor enclosure is to IP55—one level better than Newman's main competitors—the terminal box goes one step better again, and gives the added protection of IP65.

Another standard MM Series feature, usually an optional extra, is the provision of a drilled and tapped shaft.

By fitting the motor feet appropriately to three alternative pairs of mounting pads around the frame, the terminal box can be positioned to the left, on top or to the right without disconnecting the stator leads. The terminal box itself can be turned as required to give cable entry from one of four directions at 90°.

Cast-iron adaptor rings, for BS flange or face mounting are simply located on the motor end brackets and fixed by tie bolts or studs, to convert to different mounting configurations—with or without the feet. The motors can also, of course, be directly pad-mounted, as for airstream, fan motor applications.

By specification at time of ordering, the motors can be supplied to BS Standard Metric dimensions BS5000 and IEC72, or to American NEMA dimensions.

Similarly, they are available wound for 2, 4, 6 or 8 pole speeds, 50 or 60 Hz, in all standard voltages up to 660 V maximum.

Class 'F' insulation is used throughout, with ratings based on a temperature rise to class 'B' across most of the range. This gives higher thermal capacity and increased reliability, as well as the ability to cope with high ambient temperatures and above average overload or adverse supply variations.

*Details from Newman Electric Motors Ltd, Station Road, Yate, Bristol BS17 5HG, UK. Tel.: 0454 313311.*

**Circle No. 44 on Reader Enquiry Card**

### EDT research add MCI elemental analysers to their range

EDT Research has recently been appointed the UK agents for Mitsubishi Chemical Industries Ltd. MCI manufacture a range of elemental analysers which fit in directly with EDT's own range of electrochemical instrumentation. The elemental analysers include the total sulphur chlorine analyser (Model TSX-10): this microprocessor controlled instrument was developed using MCI's widely respected high-performance coulometer. It measures total chlorine or total sulphur in liquids or solids over a sensitivity range of from ppm to percentage. The model TN-02 is a total nitrogen analyser incorporating the coulometry for multiple applications. MCI developed this automated, labour-saving unit in response to the increased attention being focused on nitrogen and sulphur contents of fuel, oil and other solutions, as well as total nitrogen in water and waste water. The trace sulphur analyser (Model TS-02) covers the determination of sulphur in two ranges, 0·5 ppm–2000 ppm and less than 0·5 ppm. Sample sizes can be 1–200 µl for liquids and 1–20 ml for gases. One of the latest instruments from MCI is a total organic halogen analyser (Model TOX-10). Organic halogens in city water, especially purgeable organic halogen compounds (POX), such as trihalomethanes (THM) are suspected to have adverse effects on the human body. MCI developed this instrument in direct response to worldwide demand.

*Further information may be obtained from EDT Research, 14 Trading Estate Road, London NW10 7LU. Tel.: 01 961 1477.*

**Circle No. 45 on Reader Enquiry Card**

### Flow monitoring system

A new modular flow monitoring unit, PC50, from Control Ability Ltd, is designed as a basic dual-channel panel-mounting device for applications in fluid metering and control systems. It is capable of accepting low or high rate pulsed inputs from turbine or positive displacement transducers.

Programming of the scalar quantities involved is via a 10-digit key-pad on the front of the panel. Up to eight alarm levels can be programmed, operating either relay output channel; serial outputs are available for such recording devices as printers and chart recorders.

Stored values can be held in the memory for up to a year by means of the battery back-up. A reset button enables a batch operation to be repeated without reprogramming. A six-digit LED display can show either a total or rate of flow as required and a two-digit LED display indicates the function being performed.

Monitoring pulse rates are from 0·001 Hz to 2 kHz. Operating temperature range is 0–50 °C. Designed for DIN panel mounting, the unit measures 96 × 96 × 175 mm deep.

*More information from Control Ability Ltd, St Lawrence House, Mill Street, Great Harwood, Blackburn, Lancashire BB6 7NN, UK. Tel.: 0254 886685.*

**Circle No. 46 on Reader Enquiry Card**

### Intelligent valve positioner

Hamilton has introduced a digitally controlled valve driver and positioning system to be used with HVP, HVX and multiport valves. This product consists of a valve driver assembly (including valve, synchronous motor and encoder) and a controller unit.

The controller will drive up to four valves; it can also be cascaded to other controllers thereby operating up to 320 valves from one source. The operator can program the IVP to switch any or all of the valves, simultaneously and independently, to any valid position for the valve in any desired sequence. A standard RS 232 interface used with the controller provides field selectable baud rates of 300, 1200, 2400 and 9600. The programmable control of 12 positions satisfies the operation of Hamilton's two-, three-, four-, six and eight-port valves. Port-to-port speed is 1/4 of a second for an eight-port valve.

Applications will include:

- Multi-column switching in HPLC.
- Gradient generator for HPLC.
- Sample selection in spectrophotometry.
- Multi-reagent selector in spectrophotometry and clinical chemistry analysis.
- Automation in dissolution testing.
- Interfacing with diluters, dispensers or any other discipline where controlled flow direction is important.

*Details from Hamilton Bonaduz AG, PO Box 26, CH 7402 Bonaduz, Switzerland.*

**Circle No. 47 on Reader Enquiry Card**

### Spectroscopy method discriminates between smokers' and non-smokers' blood

A Philips Analytical PU 8800 spectrophotometer is being used by clinical chemists at a Nottinghamshire hospital to differentiate between the blood of smokers and non-smokers. The method—carried out at Kings' Mill Hospital at Sutton-in-Ashfield—is simple, straightforward to set up, and fast. Results can be obtained within 15 min of receiving a sample.

The procedure first involves converting haemoglobin to its reduced form. The magnitude of the zero-order spectral shift of the reduced haemoglobin peak at 430 nm to the carboxyhaemoglobin peak at 418 nm is then determined by second-derivative spectrum analysis, using the PU 8800 high performance scanning ultraviolet/visible spectrophotometer.

The spectrum of a compound or mixture of compounds can be mathematically differentiated to produce derivative spectra.

Odd-numbered derivatives are of most use in determining the exact points of absorbance maxima of the original or zero-order spectrum and hence the qualitative properties of the substance under investigation. Even-numbered derivatives are helpful in quantitative determinations.

The latter prove particularly useful when the properties of zero-order spectrum are changed as a result of interferents such as base-line shift, alteration in base-line slope, the presence of other compounds with similar optical properties, or of inert material such as air bubbles, insolubles or colloidal particles.

The application of derivative spectroscopy to the measurement of carboxyhaemoglobin is important because current methods are beset by problems. Zero-order spectroscopy is insensitive and confused by the presence of reduced haemoglobin, the Tietz and Fiereck procedure is also insensitive, and diffusion methods are time consuming and unsuitable for routine use.

*Further information from Pye Unicam Ltd, York Street, Cambridge CB1 2PX, UK. Tel.: 0223 358866.*

**Circle No. 48 on Reader Enquiry Card**

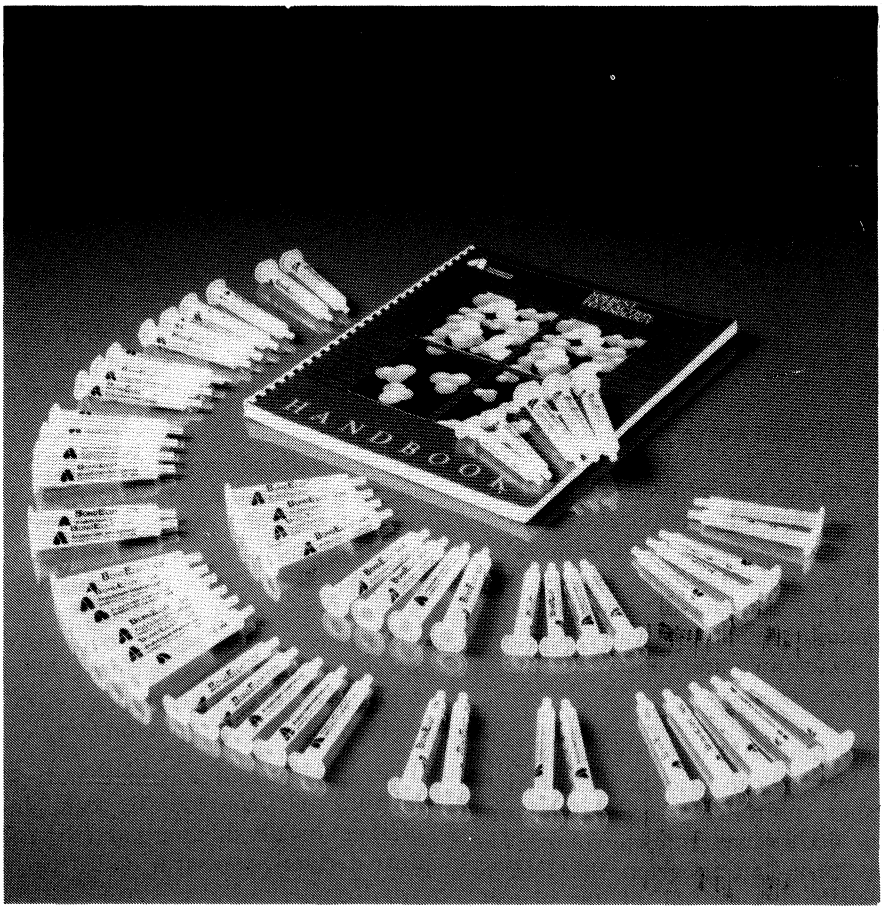

*Analytichem International's 124-page 'Sorbent Extraction Technology Handbook' explains the theory of the technology and its applications in sample preparations and chemical isolation and purification. The fully illustrated handbook shows how sorbent extraction technology is used in pharmaceutical, food, petrochemical, environmental, biomedical, and clinical applications. Information from Analytichem International, PO Box 234, Cambridge CB2 1PE, UK.*

**Circle No. 49 on Reader Enquiry Card**

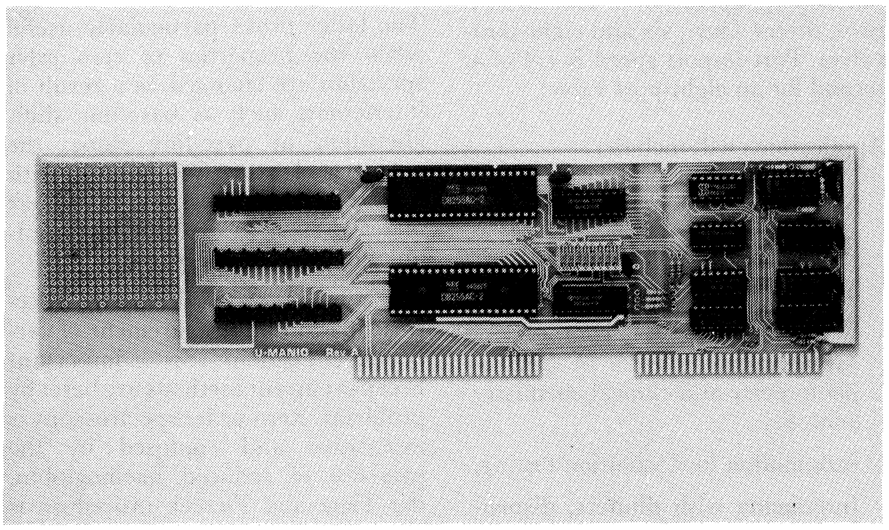

*U-Microcomputers is moving into the fast data acquisition market with a new product for the U-MAN Series 1000. The U-MANIO is a three-channel 16-bit parallel interface card with support software that can read or write a 16-bit word in 500 nano-seconds. A new version of their 12-bit A/D system is now available that has a 15 ms maximum conversion time. Full information from U-Microcomputers Ltd, Winstanley Industrial Estate, Long Lane, Warrington, Cheshire WA2 8PR, UK. Tel.: 0925 54117.*

**Circle No. 50 on Reader Enquiry Card**

### Bench-top haematology analyser

The cytochemical differential produced by the Technicon H6000 and H6010 has long been accepted to be the best automated white cell differential. The H6000 and H6010 have shown by their increasing share of the haematology analyser market to be successful in that segment of the market where their technology is most appropriate, i.e. laboratories performing more than 400 blood samples per day.

However, two areas of criticism have always been levelled at the H6000 and H6010—their cost, where workloads are lower than the optimum, and their complexity of operation—particularly for smaller laboratories where a dedicated operator may be more difficult to find.

The Technicon H1, however, addresses both these criticisms and brings within reach of the average district general hospital the sophistication of results previously only available from the H6000 and H6010.

This bench-top, discrete, selective analyser uses the cytochemical differential perfected on Technicon's other haematology systems with two important differences—the dwell time is less than 1 min and the differential is an option on each sample. Therefore the analysis of an urgent sample may be performed at any time day or night, selecting complete blood count only or complete blood count plus differential. A total picture will be displayed on the VDU and produced as a hard-copy report on both ticket printer (number only) and screen printer if desired.

The system requires a mixed EDTA blood sample from which 100 µl is aspirated. The probe is automatically cleaned and dried between samples using a suction collar. Within the analytical console is stored sufficient reagents to perform 900 samples (3 ml of reagent per sample). The blood is segmented using a ceramic shear valve prior to mixing with the appropriate reagents. The haemoglobin and white cell peroxidase estimates are performed using white light, whereas red cells, platelets and white cell basophils are measured with a laser source at two angles of scatter (2–3° and 5–15°).

This use of two angles for measurement allows much greater in-depth analysis of the cells under examination. For example, the haemoglobin content of each red cell can be directly measured using the Mie formula producing the first truly accurate automatic determination of red cell size, independent of MCHC; also the laser is able to give greater information on the white cells, producing a neutrophil lobulation index.

The net effect of this advance is a simplification of result production with the print-out reporting in the traditional way—anisocytosis, hypochromia, macrocytosis etc. as absent, +, ++ or +++.

In producing a simple-to-use system, Technicon have also addressed the problem of costs. The capital cost will be competitive with other discrete analysers performing less sophisticated white cell screens. Because of the discrete approach of the H1, reagent usage is limited only to the time of sample aspiration, thus allowing the smaller laboratory to benefit from cytochemistry without needing the continuous supply of samples which make the H6000 and H6010 cost-effective.

*More information from Technicon Instruments Ltd, Evans House, Hamilton Close, Houndmills, Basingstoke, Hampshire RG21 2YE, UK. Tel.: 0256 29181.*

**Circle No. 51 on Reader Enquiry Card**

### CHemometrical OPtimization by Simplex

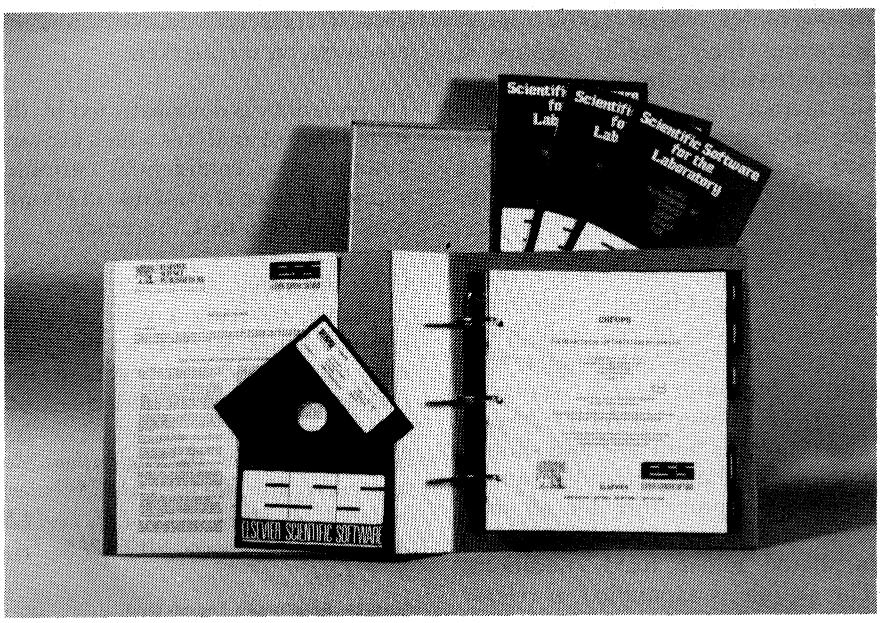

*CHEOPS is available for both the IBM-PC and Apple II series and is priced at $295. This includes the diskette, the source code listings and the complete manual. The source code listings allow the user to adapt the program to suit his own specific requirements. The authors are P. F. A. van der Wiel, B. G. M. Vandeginste and G. Kateman from the Department of Analytical Chemistry at the University of Nijmegen, The Netherlands.*

Industrial or institutional research laboratories carry out a great deal of optimization work of a very diverging nature. This may be to optimize the yield of a synthesis; maximize the output of a production plant; optimize the separation factor for two compounds in a chromatographic separation; optimize the composition of a polymer; or optimize the determination of an enzyme in blood plasma.

Optimizations can often be very tedious: many experiments may be necessary since several (frequency mutually dependent) parameters usually influence the ultimate result. Not even the conventional method of systematically varying one parameter at a time can guarantee that the optimum is found.

The software package CHEOPS offers an intelligent sequential optimization plan. It incorporates the modified and super-modified sequential simplex optimization methods. Several options are also built in (weighted centroid method, normal reflection etc.), allowing the user to tailor the optimization plan to meet his requirements. Optimizations involving variation of up to 10 parameters can be carried out with CHEOPS. As the method used searches for a short pathway to reach the optimum, the number of experiments is usually much fewer if compared with the conventional 'one factor at a time approach'.

CHEOPS comprises five programs: the main driver; an input program; the modified simplex optimization; the super-modified simplex optimization program. The input program is used to choose and define a simplex method and to specify all the parameters needed in the optimization. The output of this program is then used by one of the two simplex programs to start the optimization process. This process can be interrupted by the user at any time and can be resumed without any loss of information.

*Further information can be obtained from Keith Foley, Elsevier Scientific Software, PO Box 330, 1000 AH Amsterdam, The Netherlands. Tel.: 020 5862 828*

**Circle No. 52 on Reader Enquiry Card**

### Indentifying microbes

The number of organisms needing to be identified in the microbiology laboratory has increased remarkably over the past few years. Newly discovered species such as *Legionella pneumophila* have assumed a considerable significance, and the range of specific biochemical tests needed to identify bacteria has multiplied. The gas chromatographic procedure at the heart of the new Hewlett-Packard system (*The HP 5898A Automatic Microbial Identification System*) is not, however, limited to specific groups of bacteria. By separating the cellular fatty acids which are characteristic of the genotype, and automatically searching their profiles against a computer-based reference library, the method can be used to identify any bacterium isolated in pure culture *in vitro*. The system is limited only by the reference data held on file.

The system combines high-resolution gas-liquid chromatographic analysis of cell wall lipids, using the HP 5890A chromatograph, with a computer search against a library of known microbial strains. The process is both safe and simple, with a standard, one-tube sample preparation procedure for all microbes which are first killed, then incubated under fixed conditions. The sample is saponified to release the fatty acids, which are extracted from the aqueous phase before being subjected to chromatographic analysis. The system is calibrated automatically, and the peaks named and quantified. The computer then searches the library for the best and second-best profile matches at genus, species and (where possible) subspecies level, and a hard copy of the analysis and search results is printed out.

The complete HP 5898A system is now available in the UK at around £33 000. Hewlett-Packard can also offer a two- or three-year operating lease package, under the terms of which users will be able to rent the system for around £2800 per quarter (plus tax). This would normally come out of the day-to-day operating or revenue budget, and not out of capital funding. In use, the system identifies bacteria at a cost that compares very favourably to that of plastic strip biochemical identification, and at a fraction of the cost of conventional reference methods.

*Product enquiries to Tina Mears, Analytical Instrumentation, Hewlett-Packard Ltd, Miller House, The Ring, Bracknell, Berkshire RG12 1XN, UK. Tel.: 0344 424898.*

**Circle No. 53 on Reader Enquiry Card**

### Automated industrial control analysis

The AMICA System, developed by Hamilton of Switzerland, has been described as being one of the most cost-effective automated industrial analysers on the market.

Its versatility is demonstrated by the wide range of analyses which are now automated; a confidential customer support service is available in Hamiltons Applications Laboratory.

Over 80 applications are currently available, covering a wide range of methodologies for the water, pharmaceutical, food and beverage, textile, detergent and metal industries.

*For information on the applications that have successfully used the AMICA System, contact V. A. Howe & Co. Ltd, 12–14 St Ann's Crescent, London SW18 2LS. Tel.: 01 874 0422.*

**Circle No. 54 on Reader Enquiry Card**

### Modex has 5 amp current overload unit

A three-phase overload unit operating from standard 5 A current transformers, the OLR 600, has been announced by Welwyn Modex Automation, a member of the Crystalate Group.

Retailing at £70, it uses digital techniques to give accurate overload monitoring for three phase AC systems. The unit operates from three current transformers connected in star formation to provide approximately 4 A from each phase at full load. The OLR can also be used with the normal metering current transformers.

The unit selects the highest of the input currents, and if this exceeds the full load current setting, the full-load LED glows. When the current is 10% above full load current, the counter in the unit starts counting pulses produced by the internal circuit, whose frequency is proportional to the percentage overload.

The relay will de-energize when the counter is full. The counting system is over-ridden by the instant trip facility, which is also set on the front panel, and is scaled in multiples of full load current.

The counting system gives a response time inversely proportional to the level of the overload current. The standard timing for the OLR gives a 15 s delay at twice full load current—other timings can be arranged on request.

Specifications include a 24 V DC supply or 12 V DC supply, 3–5 A AC 3 phase full load setting, one to three times full load setting, and a relay contact of DPCO 3 A, 240 V AC maximum.

*More information from Frank Hartley, Welwyn Modex Automation, St Leonards Avenue, Hayling Island, Hampshire PO11 9BW, UK. Tel.: 0705 462893.*

**Circle No. 55 on Reader Enquiry Card**

### Fluorescence in biological analysis

*An Introduction to Fluorescence in Biological Analysis* by A. T. Rhys Williams is now available from Perkin-Elmer. Several important changes both in technology and technique have occurred recently which enhance the use of fluorescence spectroscopy in biological analysis. For example, the combination of the separative power of liquid chromatography, together with the sensitivity of fluorescence, has provided the analyst with a very powerful tool; the advent of microprocessors has increased the flexibility of instruments and at the same time improved the reliability, sensitivity and precision of measurement. This booklet will bring the reader up-to-date on the many new applications of fluorescence in biological analysis.

*To obtain a free copy write to Perkin-Elmer Ltd, Post Office Lane, Beaconsfield, Buckinghamshire HP9 1QA, UK.*

**Circle No. 56 on Reader Enquiry Card**

### Natural gas analysers brochure

Packard Instrument Ltd have introduced a 16-page brochure describing its range of natural gas analysers. Included in the brochure is a range of optional systems dependent upon which specific analysis the customer requires.

*For further details contact Packard Instrument Ltd, 13–17 Church Road, Caversham, Berkshire RG4 7AA, UK. Tel.: 0734 478234.*

**Circle No. 57 on Reader Enquiry Card**

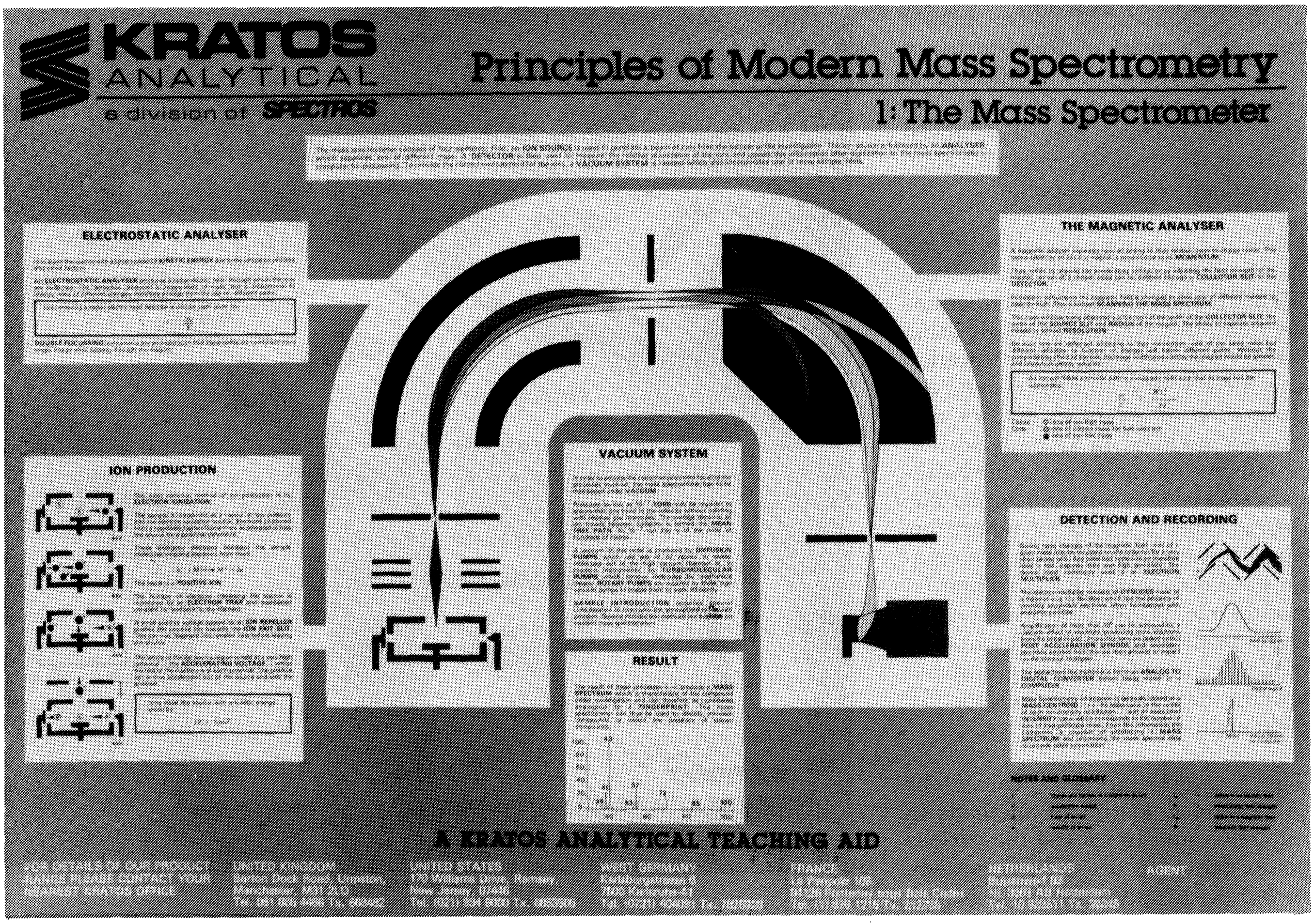

*A series of wall charts outlining the principles of mass spectrometry has been produced by Kratos Analytical. Designed as teaching aids, they are available on request to teaching hospitals, university departments and schools. The first covers the mass spectrometer itself and its four component elements. These, explains the chart, consist of an ion source to generate a beam of ions from the sample under investigation, an Analyser to separate those of different mass, a detector to measure the relative abundance of the ions and a vacuum system to provide the appropriate environment. The resulting mass spectrum, it concludes, provides identification of the original sample. These new teaching aid posters, measuring 700 × 500 mm, have been prepared by Kratos's Alan Parsons and the first is available free from him at Barton Dock Road, Urmston, Manchester M31 2LD, UK.*

**Circle No. 58 on Reader Enquiry Card**

### Chromatography work-station

Based on the HP 9000 Series 300 technical computers, the Hewlett-Packard's work-station is initially offered with the HP 1090M Series liquid chromatographs and the HP 1040M diode array detection system (the M suffix denotes that the new work-station is included in the package). Within a short time, it will also become the standard work-station for all HP gas chromatography and bench-top mass selective detector systems.

Diode array detection makes a large amount of data available to the chromatographer. Spectral and chromatographic data are acquired very rapidly—during an analysis up to 8 spectra/s can be saved, over the wavelength range 190–600 nm. The work-station is designed to handle and process this with maximum efficiency.

The good colour graphics, multiple windowing and operating simplicity of the HP 9000 Series 300 computer are put to good effect in the new workstation. During a run, the status of the entire LC system may be displayed on the screen, or the developing chromatogram and changing spectra may be viewed simultaneously.

After a run, chromatograms at any specified wavelength and bandwith may be reconstructed from the spectral data. A spectrum is displayed in the top window of the screen, and by moving a horizontal cursor across the wavelength axis, different chromatograms appear in the lower window. The wavelength offering the best sensitivity and selectivity for the compound is soon determined. Individual peaks may be enlarged for more detailed inspection.

Using a vertical cursor across the time axis, spectral changes over the run can be reviewed. Spectra from different parts of the peak may be frozen on the display, normalized and superimposed, so that individual peaks may be checked for purity. An overlay of the peak at several wavelengths appears in the same display, and any shift in retention time due to an impurity is clearly seen.

A feature of the work-station is the ability to convert all spectra from a run into a map of iso-absorbance data. The conversion is effected in seconds at the touch of a single key, and the cursors may be moved through the map to give a thorough assessment of the sample.

Once the optimum wavelength and bandwidth for each compound has been established, the chromatograms are quantitated simultaneously using integration software. All integration parameters and timed events are defined on the graphics display, and can be modified in seconds so that re-integration may be performed without the need to rerun the sample. The system gives a choice of report formats. Results are stored on Winchester hard disks. An HP 2225 ThinkJet printer may be used for instant hard-copy print-out, while presentation quality copies of chromatograms and spectra may be produced on the HP 7440 eight-pen colour plotter.

The work-station can be programmed to run its methods in sequence, and to control the various components of the HPLC system, including an autosampler to permit unattended operation. By programming the parameters for separation, detection, evaluation, integration and reporting for each LC method, totally automated analyses may be performed. Hewlett-Packard's new chromatography work-station provides an unprecedented degree of control and data manipulation and extrapolation, and is set to become the new industry standard.

*Product enquiries to Analytical Instrumentation, Hewlett-Packard Ltd, Miller House, The Ring Bracknell, Berkshire RG12 1XN, UK. Tel.: 0344 424898.*

**Circle No. 59 on Reader Enquiry Card**

### A new series of IR spectrometers from Perkin-Elmer

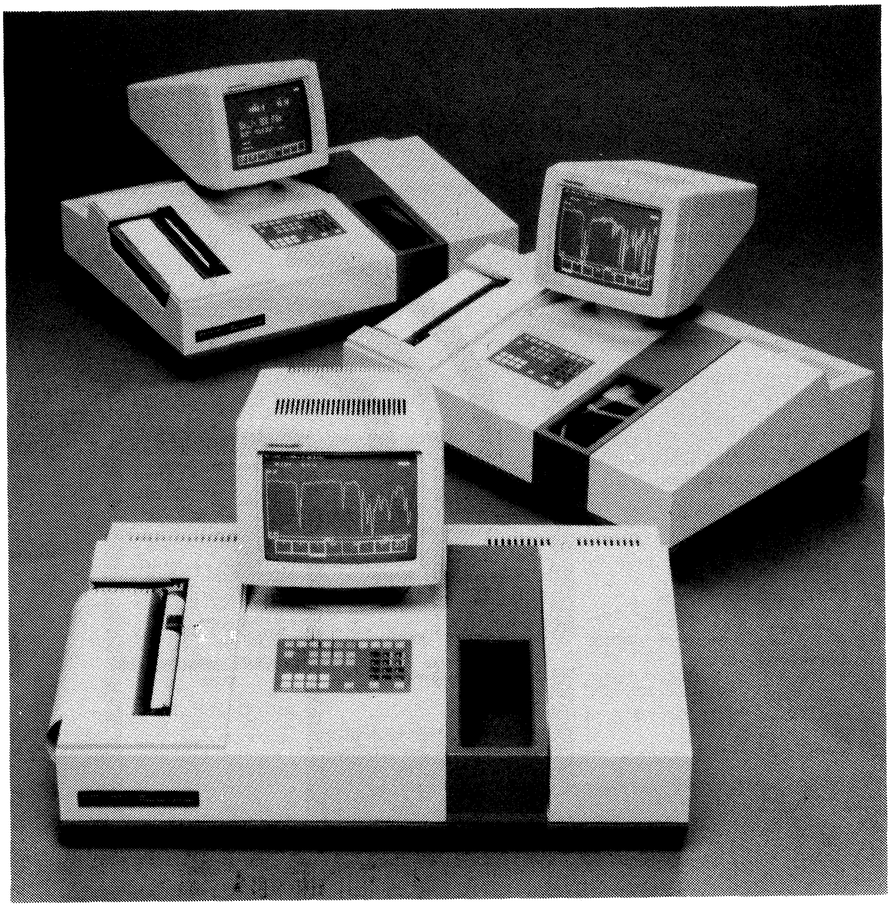

*An RS232C interface is provided with the 800 series for communication with external computers. This allows the full range of IR Application software to be used as well as giving access to the Perkin-Elmer Laboratory Management System.*

**Circle No. 60 on Reader Enquiry Card**

The new 800 Series ratio recording infra-red spectrometers from Perkin-Elmer offer high performance and spectroscopic versatility, an unprecedented number of standard data handling routines and stored methods, quantitative analysis, interactive difference, spectral memories, graphics, data formatting and data manipulation.

The series consists of three instruments: Models 881, 882 and 883, covering the ranges 4000 to 600 $cm^{-1}$, 400 $cm^{-1}$ and 200 $cm^{-1}$ respectively. A monochrome VDU in an adjustable housing provides fast, high resolution graphics allowing full- or part-range infra-red spectra to be viewed and manipulated before being plotted. Spectral data can be copied from one region to another, processed, reformatted and reset at any stage to the original spectrum so that the raw data is never lost. Hard copy output is provided by an ultra-fast digital plotter/printer. Plot and print functions permit data to be replotted or results to be printed without having to rerun the spectrum.

An entirely new optical and electronic design concept results in outstanding spectroscopic performance. Ratio recording allows very weakly absorbing samples be measured with a high signal-to-noise ratio. Fast scanning and high sensitivity allow a survey spectrum to be measured and plotted in less than a minute. Fast sampling techniques, such as diffuse reflectance, are well suited to the speed and sensitivity of the 800 Series.

*Further details from Perkin-Elmer Ltd, Post Office Lane, Beaconsfield, Buckinghamshire HP9 1QA, UK.*

**Circle No. 61 on Reader Enquiry Card**

